# Red blood cell transfusion improves microdialysis-assessed interstitial lactate/pyruvate ratio in critically ill septic patients

**DOI:** 10.1186/cc11053

**Published:** 2012-03-20

**Authors:** P Kopterides, N Nikitas, M Theodorakopoulou, A Diamantakis, D Vassiliadi, A Kaziani, S Assoti, F Drakopanagiotakis, A Antonopoulou, P Papadopoulos, E Mavrou, C Georgiadou, A Tsantes, A Armaganidis, I Dimopoulou

**Affiliations:** 1 'Attiko' University Hospital, Haidari - Athens, Greece

## Introduction

Even though red blood cell (RBC) transfusion is a common intervention in the critical care setting, there is a paucity of data regarding its impact on tissue metabolism. The aim of this study was to explore the effect of RBC transfusion on microdialysis-assessed interstitial fluid metabolic parameters in septic patients.

## Methods

We conducted an observational, clinical study in a 25-bed, medical-surgical ICU of a university hospital. We analyzed the effect of transfusion of either 1 or 2 RBC units on interstitial fluid metabolic activity by means of a microdialysis (MD) catheter inserted in the subcutaneous adipose tissue of the upper thigh. Samples were collected before (T0) and after (T1a and T1b; spaced out by 4 hours) transfusion. Lactate, pyruvate, glycerol and glucose concentrations were measured with a bedside analyzer and the lactate/pyruvate (LP) ratio was calculated automatically.

## Results

We enrolled 37 patients with severe sepsis/septic shock requiring RBC transfusion. After transfusion, the mean arterial pressure increased from 79 ± 9 to 82 ± 10 (T1a vs. T0: *P *< 0.05) and 83 ± 10 mmHg (T1b vs. T0: *P *< 0.001). Besides a nonstatistically significant drop in arterial partial oxygen pressure, we observed no change in arterial blood gases and vital signs. Overall, RBC transfusion did not alter any of the MD-assessed parameters (that is, lactate, pyruvate, glycerol and glucose) or blood lactate, but it decreased the tissue LP ratio from (T0) 18.80 (interquartile range (IQR), 14.85 to 27.45) to (T1a) 17.80 (IQR, 14.35 to 25.20) (*P *< 0.05) and (T1b) 17.90 (IQR, 14.45 to 22.75) (*P *< 0.001). The post-transfusion changes in LP ratio at T1a (*r *= -0.42; 95% CI, -0.66 to -0.098; *P *= 0.01) and T1b (*r *= -0.68; 95% CI, -0.82 to -0.44; *P *< 0.001) were significantly correlated with the pre-transfusion LP ratio but not with baseline demographic characteristics, vital signs, severity scores, hemoglobin level and blood lactate. Finally, 39.0% of the transfused RBC units were leukoreduced and their median storage time was 16 days (IQR, 11 to 24). RBC storage time and leukocyte reduction had no influence on the tissue metabolic response to transfusion.

## Conclusion

Tissue oxygenation is improved by red blood cell transfusion in critically ill septic patients. Monitoring of the tissue LP ratio by microdialysis may represent a useful method for individual clinical management.

